# The combined effect of cognitive impairment and pain on functional and mobilitydisability among older adults in India

**DOI:** 10.1002/alz.087507

**Published:** 2025-01-09

**Authors:** Thalil Muhammad, Manacy Pai

**Affiliations:** ^1^ Pennsylvania State University, State College, PA USA; ^2^ Kent University, Kent, OH USA

## Abstract

**Background:**

We examined the associations between cognitive impairment, experiences of pain, and functional and mobility disability among older adults in India. Moreover, we assessed the combined effects of cognitive impairment and pain on functional and mobility disability among older Indians.

**Method:**

Data come from baseline wave (2017‐19) of the Longitudinal Aging Study in India, with a sample of 31,464 older adults aged 60+ years. Cognitive impairment was assessed using measures of memory, orientation, arithmetic function, and visuo‐spatial construction skills. Functional and mobility disability was evaluated using the scales of activities of daily living (ADLs, score 0‐6), instrumental activities of daily living (IADLs, score 0‐7), and mobility (score 0‐9). Pain and pain frequency was self‐reported. Multivariable linear regression models were employed to examine the association between variables, and the interaction terms were used to test the moderation effects.

**Result:**

Older adults who were cognitively impaired were more likely to report ADL (beta: 0.295, CI: 0.238, 0.352), IADL (beta: 0.642, CI: 0.547, 0.737), and mobility disability (beta: 0.605, CI: 0.490, 0.719). Similarly, those reporting any pain were more likely to report ADL (beta: 0.258, CI: 0.224, 0.292), IADL (beta: 0.421, CI: 0.367, 0.475), and mobility disability (beta: 1.320, CI: 1.244, 1.396). Further, the interactions between cognitive impairment and pain were statistically consequential (p<0.001) for ADL, IADL, and mobility disability, and the associations between cognitive impairment and functional and mobility disability were more pronounced among older women and those experiencing frequent pain (5+ days/week).

**Conclusion:**

Our findings underscore the need for integrated healthcare approaches that address cognitive health and pain management simultaneously to help older adults maintain functional abilities and mobility. That female gender and frequent pain magnify the association between cognitive impairment and functional and mobility disability is useful information for providers and practitioners offering individualized care plans to older patients.

